# Development of a 10-km resolution global soil profile dataset for crop modeling applications

**DOI:** 10.1016/j.envsoft.2019.05.012

**Published:** 2019-09

**Authors:** Eunjin Han, Amor V.M. Ines, Jawoo Koo

**Affiliations:** a International Research Institute for Climate and Society, Columbia University, NY, 10964, USA; b Department of Plant, Soil and Microbial Sciences, Michigan State University, Michigan State University, MI, 48824, USA; c Department of Biosystems and Agricultural Engineering, Michigan State University, MI, 48824, USA; d International Food Policy Research Institute, Washington, DC, 20005, USA

**Keywords:** Global gridded-soil profile dataset, Crop simulation modeling, DSSAT

## Abstract

One major challenge in applying crop simulation models at the regional or global scale is the lack of available global gridded soil profile data. We developed a 10-km resolution global soil profile dataset, at 2 m depth, compatible with DSSAT using SoilGrids1km. Several soil physical and chemical properties required by DSSAT were directly extracted from SoilGrids1km. Pedo-transfer functions were used to derive soil hydraulic properties. Other soil parameters not available from SoilGrids1km were estimated from HarvestChoice HC27 generic soil profiles. The newly developed soil profile dataset was evaluated in different regions of the globe using independent soil databases from other sources. In general, we found that the derived soil properties matched well with data from other soil data sources. An ex-ante assessment for maize intensification in Tanzania is provided to show the potential regional to global uses of the new gridded soil profile dataset.

## Highlights

• Global gridded-soil profile dataset at 10-km resolution was developed for DSSAT crop simulation models.
• Required soil properties were derived from SoilGrids1km, well-proven pedo-transfer functions and HarvestChioce's HC27.
• The developed soil dataset was evaluated in comparison with other published soil databases.
• The dataset opens potential applications of DSSAT for ex-ante assessments at the regional or global scale.

## 1 Introduction

The needs for assessing impacts of global environmental change on agriculture and food security stimulated the use of process-based crop models at the regional to global scales (Basso et al., 2018; Challinor et al., 2010; Jones et al., 2017; Nelson et al., 2010; Rabbinge and Van Diepen, 2000; Rosenzweig et al., 2014; Rosenzweig et al., 2013; Wu et al., 2010; Xiong et al., 2008). Crop models have been widely used also in developing early warnings and decision support systems (Asfaw et al., 2018; Gyawali et al., 2018). Despite the known issues of scaling up point-scale processed-based crop models onto larger spatial scales (Faivre et al., 2004; Hansen and Jones, 2000), they have been applied at larger scales beyond the spatial footprint, which they were initially developed. This is because crop models allow us to better understand the dynamic interactions between crops and the changing environment, which statistical models are limited from doing.

Applications of well-validated process-based crop models at the regional or global scale require a gridded framework (Elliott et al., 2014, 2015; Folberth et al., 2013; Liu, 2009). Considering that a complex crop model developed at a field-scale requires a considerable amount of data inputs including climate, soil and crop management information, a gridded crop modeling framework requires even more information with spatial consistency, and it is inevitable to make assumptions at the aggregate scales. Among others, soil data are particularly problematic because it is difficult and expensive to estimate representative soil properties at the aggregate scale through field surveys (Lagacherie et al., 2000). While climate data have been increasingly available from various sources that include those from weather stations, remote sensing and reanalysis data, the availability of spatially contiguous gridded soil datasets in a crop model specific format is still a major challenge for gridded crop simulations (Wu et al., 2010). There is a critical need to develop practical solutions to bridge this gap.

To date, there have been several efforts in the development of global soil databases. To name a few: Harmonized World Soil Database (HWSD) (Nachtergaele et al., 2008), International Soil Reference and Information Centre (ISRIC)–World Inventory of Soil Emission Potentials (WISE) derived soil properties (Batjes, 2012) and recently, ISRIC - World Soil Information Service (WoSIS) (Ribeiro et al., 2015). These soil databases were converted into three-dimensional digital soil maps by interpolating field measurements with other environmental covariates (Hengl et al., 2014; Sanchez et al., 2009). The resulting digital soil maps are published in a standard format, grid-based, and at high resolution. For example, Hengl et al. (2017) and Hengl et al. (2014) developed global gridded soil maps at 250-m and 1-km resolution respectively. These digital soil maps, however, cannot be readily used in crop models because most process-based models require more soil information than what these soil databases provide, and soil input must be tailored to with the model's specific format.

Crop models in the Decision Support System for Agro-technology Transfer, DSSAT (Hoogenboom et al., 2017; Jones et al., 2003) have been used in several global gridded crop model inter-comparisons for assessing climate change risks in agriculture (Elliott et al., 2014, 2015; Rosenzweig et al., 2013, 2014). DSSAT has been integrated in IMPACT model for policy analysis by the International Food Policy Research Institute (Robinson et al., 2015). DSSAT is also used by HarvestChoice (http://harvestchoice.org/) to simulate scenarios for improving livelihoods of smallholder farmers in sub-Saharan Africa (e.g., Rosenzweig et al., 2014). The main motivation of the present study was to develop soil inputs readily available for gridded DSSAT applications at a high resolution, 5 arc-minute (∼10 km), which is a target resolution of HarvestChoice's analysis system. We envision that the new global soil profile dataset can be used by other gridded modeling systems, thereafter. Note that, previous works for gridded DSSAT applications were done at coarser resolutions.

Based on ISRIC-WISE database, Gijsman et al. (2007) and Romero et al. (2012) generated a global point-scale soil profile dataset (WI.SOL) compatible with DSSAT crop models. WI.SOL has a very sparse soil profile data especially in the developing countries. For example, only one soil profile is available in Ethiopia. On the other hand, ISRIC's global Soil Information System called “SoilGrids” provides gridded soil properties at various resolutions from 250-m to larger scales 1-km (Hengl et al., 2017). SoilGrids opened up an opportunity to develop a gridded soil input for DSSAT.

In this study, we developed a 10-km resolution gridded global soil profile dataset compatible with DSSAT from SoilGrids datasets (datasets made in April 2015 at 1 km resolution). We evaluated the newly developed gridded soil profile dataset using several soil data from independent sources. Then, we introduced a maize intensification assessment study in Tanzania to showcase the utility of the newly developed gridded soil profile dataset. Note that our target spatial resolution (10-km) was determined to be seamlessly ingested with HarvestChoice's on-line database called CELL5M-SSA, which contains hundreds of biophysical and socio-economic indicators for Sub-Saharan Africa that enables analyses of spatial relationships between climate, environment, agriculture, nutrition, poverty and health (HarvestChoice and Minnesota, 2018).

## 2 Data

### 2.1 ISRIC SoilGrids

Recently, the international community has paid an increasing attention to improving legacy soil data resources in support of sustainable development (Folberth et al., 2016; Montanarella and Vargas, 2012; Sanchez et al., 2009). In order to contribute to the Global Soil Partnership initiative (Montanarella and Vargas, 2012), ISRIC, in collaboration with several international agencies, has developed a gridded global soil database, called SoilGrids1km, a global 3D soil information system at 30 arc second (∼1 km) resolution (Hengl et al., 2014). SoilGrids1km provides representative soil properties at six standard depth intervals specified by the GlobalSoilMap.net project, 0–5, 5–15, 15–30, 30–60, 60–100 and 100–200 cm. The soil properties include soil organic carbon (g kg^−1^), soil pH, sand, silt and clay fractions (%), cation exchange capacity (cmol kg^−1^), bulk density (kg m^−3^), coarse fragments (%), soil taxonomy based on the World Reference Base classification system and USDA soil taxonomy suborders. After compiling multiple major international soil profile databases and selecting global environmental covariates reflecting soil formation factors, Hengl et al. (2014) developed global spatial prediction models (2D or 3D regression and/or regression-kriging) to derive these soil properties. The SoilGrids1km data used in this study was the data published in 2015 (http://soilgrids.org). A newer version of the dataset called SoilGrids250 m has now been released since then (Hengl et al., 2017). The 250-m product uses machine learning, unlike the 1-km product, which used regression kriging. All of our discussions here however are all based on the 2015 SoilGrids1km. The SoilGrids250 m data will be treated in a separate study.

### 2.2 HarvestChoice HC27

HarvestChoice developed generic/proto-typical soil profiles, called HC27, which classifies soil profiles by only three criteria: soil texture, organic carbon content (as a proxy for soil fertility) and soil rooting depth (as a proxy for soil water availability) as shown in Table 1 (HarvestChoice, 2010; Koo and Dimes, 2010). HC27 aims to help overcome the limitation of traditional location-specific soil database such as WISE or HWSD when gridded simulations are needed, and to make it possible to apply crop simulation models in larger areas where detailed soil information is not available, particularly in Africa. HC27 provides 27 different soil profiles in a DSSAT-compatible format. In this study, HC27 was used to derive soil characteristics not available from the SoilGrids1km (see Section 3).

**Table 1:** Decision tree of HarvestChoice HC27 soil classification (Koo and Dimes, 2010).

Texture	Fertility	Depth	Soil Profile
Clay	High	Deep	HC_GEN0001
		Medium	HC_GEN0002
		Shallow	HC_GEN0003
	Medium	Deep	HC_GEN0004
		Medium	HC_GEN0005
		Shallow	HC_GEN0006
	Low	Deep	HC_GEN0007
		Medium	HC_GEN0008
		Shallow	HC_GEN0009
Loam	High	Deep	HC_GEN0010
		Medium	HC_GEN0011
		Shallow	HC_GEN0012
	Medium	Deep	HC_GEN0013
		Medium	HC_GEN0014
		Shallow	HC_GEN0015
	Low	Deep	HC_GEN0016
		Medium	HC_GEN0017
		Shallow	HC_GEN0018
Sand	High	Deep	HC_GEN0019
		Medium	HC_GEN0020
		Shallow	HC_GEN0021
	Medium	Deep	HC_GEN0022
		Medium	HC_GEN0023
		Shallow	HC_GEN0024
	Low	Deep	HC_GEN0025
		Medium	HC_GEN0026
		Shallow	HC_GEN0027

## 3 Methodology

### 3.1 Estimating soil properties for DSSAT

**Fig. 1:**
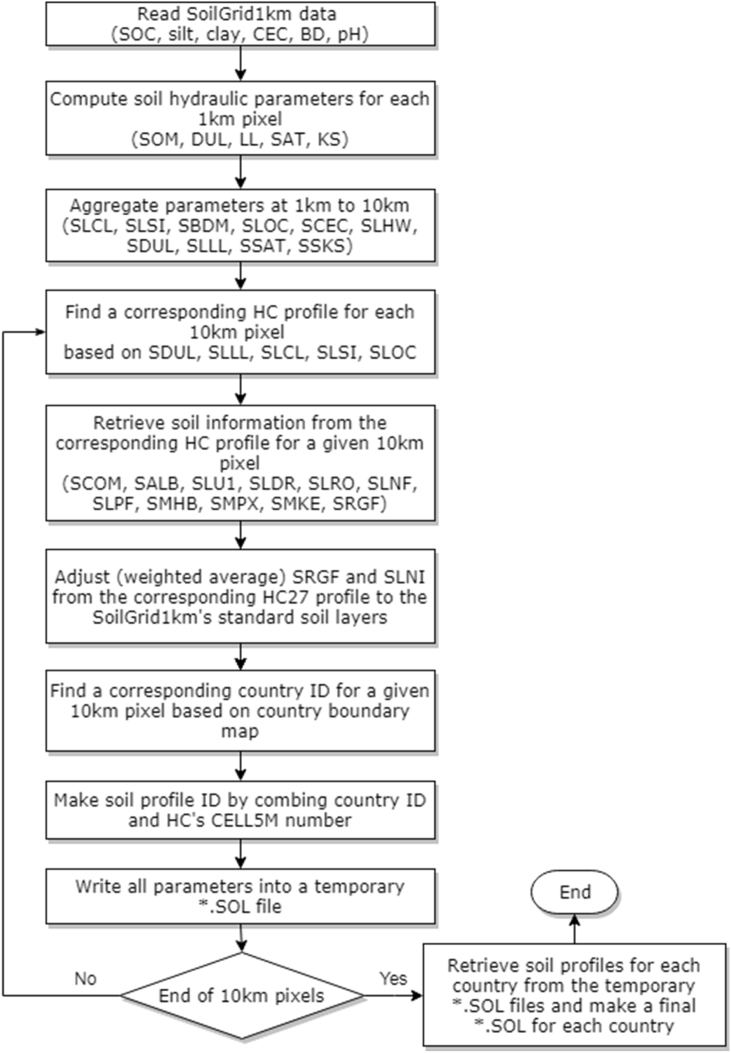
Workflows for processing and deriving soil parameters for DSSAT soil input file.

**Figure A.1:**
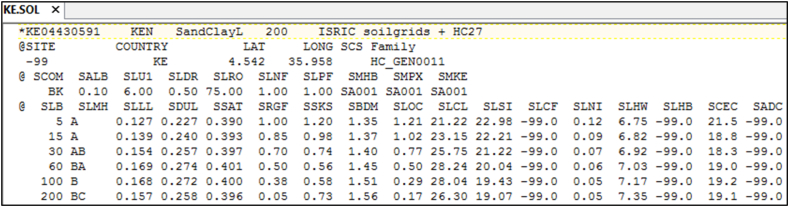
Example *.SOL output for Kenya

Table 2 shows the soil physical and chemical properties required by DSSAT. The soil data must be written in a specific format shown in Fig. A1 (Appendix). If not available, and not an essential soil property, DSSAT will fill in a missing data with a default value during run-time. In this study, soil horizons were patterned using the six standard layers of SoilGrids1km. Although SoilGrids1km provides some of the essential soil properties for DSSAT, e.g., bulk density (SBDM), organic carbon (SLOC) and fraction of silt (SLSI) and clay (SLCL), the remaining parameters in Table 2 must be estimated. The third column in Table 2 shows how we estimated each soil property. General workflow for processing SoilGirds1km and deriving soil properties for DSSAT input file (*.SOL) is shown in Fig. 1. The parameters in the first 10 rows of Table 2 (from SCOM to SMKE) inherit their values from corresponding HC27 soil profiles determined by soil properties from SoilGrids1km. Detailed procedures for deriving values of other soil properties are described in the following sections. More technical details on data processing and formatting can be found in the Appendix, and Han et al. (2015).

**Table 2:** Definitions of soil parameters in DSSAT soil file (a.SOL) (after Gijsman et al., 2007) and their estimation method.

Variable name	Definition	Unit	Method of estimation
SCOM	Soil color (Munsell color system)	–	HC27
SALB	Albedo	–	HC27
SLU1	Evaporation limit	mm	HC27
SLDR	Drainage rate	fraction day^−1^	HC27
SLRO	Runoff curve number	–	HC27
SLNF	Mineralization factor	0–1 scale	HC27
SLPF	Photosynthesis factor	0–1 scale	HC27
SMHB	pH in buffer determination method	–	HC27
SMPX	Extractable phosphorus determination code	–	HC27
SMKE	Potassium determination method	–	HC27
SLMH	Master horizon	–	Fixed ('A’, ‘A’, ‘AB','BA','B','BC')
SLLL	Lower limit of plant extractable soil water, or wilting point	cm^3^ cm^−3^	PTF (Saxton and Rawls, 2006)
SDUL	Drained upper limit, or field capacity	cm^3^ cm^−3^	PTF (Saxton and Rawls, 2006)
SSAT	Saturated upper limit	cm^3^ cm^−3^	PTF (Saxton and Rawls, 2006)
SRGF	Root growth factor	0–1 scale	Based on available water content (AWC) and HC27
SSKS	Saturated hydraulic conductivity	cm h^−1^	PTF (Saxton and Rawls, 2006)
SBDM	Bulk density (moist)	g cm^−3^	SoilGrids1km
SLOC	Soil organic carbon concentration	%	SoilGrids1km
SLCL	Clay (<0.002 mm)	%	SoilGrids1km
SLSI	Silt (0.05–0.002 mm)	%	SoilGrids1km
SLCF	Coarse fraction (>2 mm)	%	Fixed as ‘-99’^a^
SLNI	Total nitrogen concentration	%	Based on AWC and HC27
SLHW	pH in water	–	SoilGrids1km
SLHB	pH in buffer	–	Fixed as ‘-99’^a^
SCEC	Cation exchange capacity	cmol(+) kg^−1^	SoilGrids1km

a Note: The number, −99, indicates a missing value and the variables with −99 are determined by model default values.

#### 3.1.1 Estimation of the soil hydraulic properties

Soil hydraulic properties are important soil data inputs needed to run any crop models. The soil hydraulic properties determine its capacity to hold and release water needed in the calculation of the soil water balance. In order to simulate soil water movement, DSSAT requires minimum inputs to characterize soil hydraulic properties e.g., soil water content at field capacity (SDUL), at wilting point (SLLL) and saturation (SSAT) (Gijsman et al., 2002). These soil properties can be measured in the laboratory and the field, but it is practically impossible to measure them at the aggregate scale (Gijsman et al., 2002). At the aggregate scale, indirect ways of estimating these properties are essential. Many efforts have been done to derive hydraulic soil properties using more easy-to-measure properties, such as texture or proportion of sand, silt and clay. Several pedo-transfer functions (PTFs) have been developed to relate soil hydraulic properties and other soil physical and chemical properties. Rawls et al. (1991) and Timlin et al. (1996) reviewed more than 49 PTFs. There are several approaches used for developing PTFs including multiple regression, physico-empirical models and estimating parameters of equations to relate soil water contents with soil water potentials (Gijsman et al., 2002). Some studies showed that their PTFs performed well (e.g., Wösten et al., 2013). However, Gijsman et al. (2002) noted that it is difficult to recommend a certain well-performing PTFs due to big discrepancies between methods in estimating water-retention parameters. Nonetheless, they found that the PTFs of Saxton et al. (1986) performed better compared with other 7 PTFs they examined in terms of estimating field capacity, wilting point and available water holding capacity for certain soil types in the U.S. In addition, Romero et al. (2012) adapted the PTF approach by Saxton et al. (1986) to estimate soil hydraulic properties for the reanalysis of ISRIC-WISE soil database and for the development of DSSAT-compatible soil input file, WI.SOL. However, very sandy or very clayey soils were excluded from the method of Saxton et al. (1986) and Rawls et al. (1982).

In this study, we used the PTFs in Saxton and Rawls (2006), which updated the equations of Saxton et al. (1986) based on the USDA soil database, such that soil hydraulic properties can be estimated using texture and organic matter content (Table 3). Soil texture can be directly obtained from SoilGrids1km. Organic carbon from SoilGrids1km was converted to organic matter to be ingested in the equations as shown in Table 3. We multiplied a factor of 2 to the organic carbon to estimate the organic matter content. Pribyl (2010) concluded that conversion factor 2 is more accurate than the conventional value of 1.724 to convert soil organic carbon to soil organic matter.

**Table 3:** Equations used to estimate soil hydraulic properties (excerpt from Table 1 in Saxton and Rawls (2006)).

Variable	Equation	Eq.
${\text{θ}}_{1500}$	${\text{θ}}_{1500}={\text{θ}}_{1500\text{t}}+\left(0.14\times {\text{θ}}_{1500\text{t}}-0.02\right)$${\text{θ}}_{1500\text{t}}=-0.024\cdot \text{S}+0.487\cdot \text{C}+0.006\cdot \text{OM}+0.005\left(\text{S}\times \text{OM}\right)-0.013\left(\text{C}\times \text{OM}\right)+0.068\left(\text{S}\times \text{C}\right)+0.031$	i
${\text{θ}}_{33}$	${\theta_{33}=\theta_{33t}+[1.283\cdot (\theta_{33t})}^{2}-0.374\cdot \theta_{33t}-0.015]$${\text{θ}}_{33\text{t}}=-0.251\cdot \text{S}+0.195\cdot \text{C}+0.011\cdot \text{OM}+0.006\left(\text{S}\times \text{OM}\right)-0.027\left(\text{C}\times \text{OM}\right)+0.452\left(\text{S}\times \text{C}\right)+0.299$	ii
${\text{θ}}_{\text{S}-33}$	${\text{θ}}_{\text{S}-33}=\theta_{(S-33)t}+(0.636\cdot \theta_{(S-33)t}-0.107)$${\text{θ}}_{\left(\text{S}-33\right)\text{t}}=0.278\cdot \text{S}+0.034\cdot \text{C}+0.022\cdot \text{OM}-0.018\left(\text{S}\times \text{OM}\right)-0.027\left(\text{C}\times \text{OM}\right)-0.584\left(\text{S}\times \text{C}\right)+0.078$	iii
${\text{θ}}_{\text{S}}$	${\text{θ}}_{\text{S}}={\text{θ}}_{33}+{\text{θ}}_{\left(\text{S}-33\right)}-0.097\cdot \text{S}+0.043$	iv
${\text{K}}_{\text{S}}$	${{\text{K}}_{\text{S}}=1930\left({\text{θ}}_{\text{S}}-{\text{θ}}_{33}\right)}^{\left(3-\text{λ}\right)}$	v
λ	λ=1/B	vi
B	$\text{B}=\left[\ln\left(1500\right)-\ln\left(33\right)\right]/\left[\ln\left({\text{θ}}_{33}\right)-\ln\left({\text{θ}}_{1500}\right)\right]$	vii
* Definitions of symbols		
${\text{θ}}_{1500}$	−1500 kPa moisture, %v	
${\text{θ}}_{1500\text{t}}$	−1500 kPa moisture, first solution, %v	
${\text{θ}}_{33}$	−33 kPa moisture, normal density, %v	
${\text{θ}}_{33\text{t}}$	−33 kPa moisture, first solution, %v	
SAT	Saturated moisture (0 kPa), %v	
${\text{θ}}_{\left(\text{S}-33\right)\text{t}}$	SAT-33 kPa moisture, first solution, %v	
${\text{θ}}_{\left(\text{S}-33\right)}$	SAT-33 kPa moisture, normal density, %v	
${\text{θ}}_{\text{S}}$	Saturated moisture (0 kPa), normal density, %v	
S	Sand, %w	
C	Clay, %w	
OM	Organic matter, %w	
${\text{K}}_{\text{S}}$	Saturated conductivity (matric soil), mm h^−1^	
λ	Slope of logarithmic tension-moisture curve	
B	Coefficients of moisture-tension	

Even though we selected the best available PTFs for this study, one should be aware about the limitations of PTFs. Saxton and Rawls (2006) developed their equations based on USDA soil database, and there may be regions in the world where the equations will not perform well. Most studies evaluated certain PTFs by comparing results with field- or laboratory-measured soil water retention data, however “wilting point” or “field capacity” measured from laboratory may not be always applicable at the field or larger scales (Gijsman et al., 2002).

Once these fractions of sand and clay, and organic matter content are determined, the soil water content at wilting point (SLLL), field capacity (SDUL) and saturation (SSAT), and saturated hydraulic conductivity (SSKS) can be computed using the PTFs in Table 3 at the SoilGrids1km resolution.

#### 3.1.2 Estimation of the soil root growth factor

Soil root growth factor (SRGF) is an important parameter in simulating crop growth because it affects the potential rooting depth and root density of plants, which determines plants’ water uptake and eventually biomass accumulation. In spite of its importance, there is no concrete guideline to estimating SRGF (Ma et al., 2009). Because it is an empirical parameter, SRGF was often calibrated arbitrarily to improve the predicted soil water content or crop yield (Calmon et al., 1999; Fang et al., 2008). Leenaars et al. (2018) derived spatially coherent rootable depth in sub-Saharan Africa for maize by parameterizing several soil factors with a rootability index. They also argued that the absence of observed rootability or rootable depth data hindered a quantitative validation of the rootable depths map.

In this study, SRGF is estimated based on the available water storage capacity (AWC) and the corresponding soil profile in HC27. AWC is defined as the difference between water content at field capacity and wilting point, as follows:

(1) AWC = 1000(SDUL - SLLL) Z_r_

where AWC is total available soil water in the root zone (mm), SDUL the water content at field capacity (m^3^ m^−3^), SLLL the water content at wilting point (m^3^ m^−3^), Z_r_ the rooting depth (m). The FAO's HWSD classified the soil units based on AWC in 1 m soil depth as shown in Table 4 (Nachtergaele et al., 2008). Therefore, once a soil texture is known, the AWC (mm m^−1^) of the soil can be a good indicator of a possible soil rooting depth (e.g., deep or shallow). HarvestChoice used the AWC information to determine a soil as deep, medium or shallow as shown in Table 4. We also adapted a similar approach to determine a soil rootable depth from AWC and soil texture from Table 4. Once the soil depth, soil fertility and texture are determined for a target SoilGrids1km grid, a corresponding HC27 soil profile can be selected. Then SRGF of the selected HC27 soil type is assigned as the SRGF of that target pixel.

**Table 4:** Soil rooting depth identification method based on available water content (AWC).1)Compute AWC of 1 m soil depth for each SoilGrids1km grid using Eq. (1), based on the derived soil water content at field capacity and wilting point.2)Select an appropriate soil rooting depth (deep, medium or shallow) from Table 4 using the computed AWC from Step 1 and soil texture of the target 1 km pixel. For example, if computed AWC equals to 140 mm for top 1 m soil and soil is classified as clay, soil rooting depth of the target pixel is determined as “medium”.3)Based on the soil rooting depth in Step 2, soil texture and fertility of the target pixel, select a corresponding HC27 soil profile name from Table 1.

HWSD (FAO)		HarvestChoice			Present study		
Class	AWC^₊^ [mm m^−1^]	Clay	Loam	Sand	Clay	Loam	Sand
1	>150	D or M			D	D	D
2	125–150				M	M	D
3	100–125	S	D or M		S	M	M
4	75–100		S	D or M	S	S	M
5	50–75			S	S	S	S
6	15–50				S	S	S
7	0–15				S	S	S

*Note: D, M and S represent “deep”, “medium”, and “shallow” respectively.

₊Note: Available water content (AWC) applies only for the top 1-m soil depth.

Procedures for determining SRGF for a target 1-km pixel are summarized as follows.

4) Assign SRGF for each soil layer from the corresponding HC27 soil profile. Since soil depth specifications in HC27 are 0–10, 10–30, 30–60, 60–90, 90–120, 120–150, 150–180 cm (as opposed to 0–5, 5–15, 15–30, 30–60, 60–100 and 100–200 cm of SoilGrids1km), different definitions of soil depths for shallow/medium/deep and weighted averages of SRGFs are allocated to the standard SoilGrids1km layers as shown in Table 5.

**Table 5:** Example distribution of soil root growth factor for a given SoilGrids1km and HC27 soil profile.

Soil layers of SoilGrids1km (cm)	Estimation of soil root growth factor (SRGF)
0–5	HC27's first (0–10 cm) layer
5–15	Weighted average of HC27's first two layers (0.5 × SRGF of 10 cm layer + 0.5 × SRGF of 30 cm layer)
15–30	HC27's second (10–30 cm) layer
30–60	HC27's third (30–60 cm) layer
60–100	Weighted average of HC27's 4th and 5th layers (0.75 × SRGF of 90 cm layer + 0.25 × SRGF of 120 cm layer)
100–200	Weighted average of HC27's 5th, 6th, and 7th layers (0.2 × SRGF of 120 cm layer + 0.3 × SRGF of 150 cm layer + 0.5 × SRGF of 180 cm layer)

### 3.2 Evaluation of the gridded soil profile dataset at 10-km scale

Evaluating the accuracy and quality of the gridded soil dataset at 5 arc-minute resolution (10-km) as introduced in this study is not straightforward. The new gridded soil profile dataset contains soil parameters derived from multiple sources. Uncertainties of the parameters can come from many factors, e.g., measurement errors incurred in the original survey data by local agencies, standardization/harmonization process by compiling all available measurements, and spatial predictions by statistical models and aggregation to target resolutions (Heuvelink, 2014). Issues with accuracy assessment of digital soil mapping have been addressed in several studies (Bishop et al., 2015; Heuvelink, 2007; Leopold et al., 2006). Typical approach to evaluate the quality of model-predicted soil parameters is to compare them with independent point observations. However, as Heuvelink (2007) and Leopold et al. (2006) argued that differences in measurement “supports” between point observations and model predictions at larger scales should be taken into account. Bishop et al. (2015) used three different spatial supports (point, 48 m blocks and soil-land use complexes) in order to evaluate model-predicted clay content maps, and concluded that validating digital soil maps at the point support is likely to be the worst case. They argued that various spatial supports ranging from a point to larger block supports are required for better validation of digital soil maps.

In this study, the target resolution of the soil dataset is 10-km. It is difficult to have an independent data at a similar scale/support for evaluating the output soil dataset for the world. The best possible database of global coverage is the ISRIC – WoSIS (Ribeiro et al., 2015). However, WoSIS comprises point profile observations thus not free from the issue of scale/support discrepancy with our target resolution. Moreover, few point observations that fall within the target grid (10 km) is not enough to aggregate to make an average representation of the target resolution. Despite all of these, we took WoSIS as one of the validation data, and compared our derived soil hydraulic properties at 10-km resolution. The effect of different scales will be discussed in Section 4.

Due to the scale issue described above, the evaluation of the new soil profile dataset relied only on visual comparisons among other datasets in different regions of the globe. The derived soil hydraulic properties were evaluated using similar maps from literature (e.g., Leenaars et al., 2018), other soil database (i.e., SSURGO of the U.S.) and crop land distributions (Fritz et al., 2015). Regardless of these efforts, it must be reiterated that there is no “true” value to quantitatively assess the quality of the derived soil profile dataset. We can only speculate based on logic.

Furthermore, a crop simulation study is presented in Section 4.2, which showed the application of the output soil profile dataset in modeling crop yield responses over Tanzania. It can be considered an indirect way of evaluating the quality of our output soil dataset. Gijsman et al. (2007) and Romero et al. (2012) tested the sensitivities of the soil parameters they developed using crop simulation results without quantitative comparisons from other soil data sources.

## 4 Results

The final 10-km resolution gridded soil profile dataset formatted in DSSAT soil input file (*.SOL) were created separately for each 225 countries with its unique ISO_A2 as a filename (e.g., KE.SOL for Kenya). The dataset is accessible and freely available from https://doi.org/10.7910/DVN/1PEEY0 (IRI et al., 2018). The generated soil profile dataset and some applications are described below. Here, we only show some representative soil parameters as a way of evaluating the quality of the new, model friendly, soil profile dataset.

### 4.1 Derived soil hydraulic properties

#### 4.1.1 Soil water contents at wilting point and field capacity

**Fig. 2:**
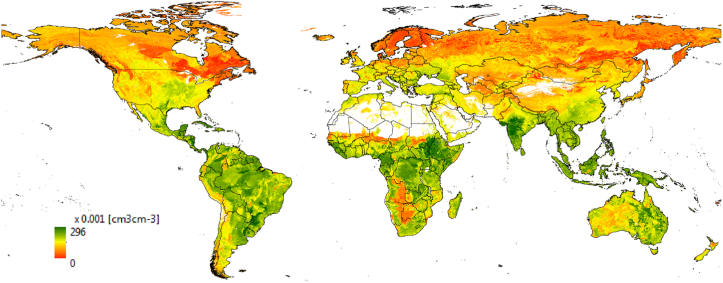
Derived soil water content at wilting point (SLLL) for 15 cm soil depth.

**Fig. 3:**
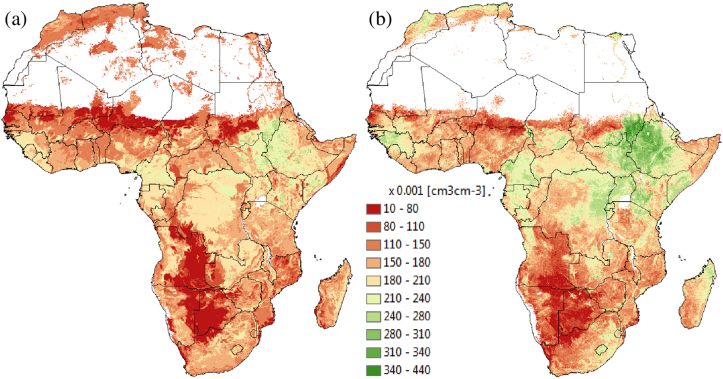
Comparison of soil water content at wilting point (SLLL): (a) derived from SoilGrids1km using a pedo-transfer function (aggregated over the top 15 cm) (b) defined at pF 4.2 from AfSIS-GYGA functional soil information for Sub-Saharan Africa (RZ-PAWHC-SSA, aggregated over the top 30 cm).

Maps of soil water content at wilting point (SLLL) at 10-km resolution are shown in Fig. 2, Fig. 3. Fig. 2 shows a depth-aggregated SLLL for the top 15 cm depth (i.e., weighted average of the first two layers) for the world. In order to evaluate the quality of the estimated SLLL, we compared the derived soil property for Africa with a permanent wilting point map from AfSIS-GYGA functional soil information for Sub-Saharan Africa (RZ-PAWHC-SSA) (Fig. 3). The AfSIS-GYGA data was introduced by Leenaars et al. (2018) and is available from www.isric.org. We used a map, moisture content (volumetric %) of the soil fine earth at permanent wilting point defined at pF 4.2 and aggregated over the top 30 cm of the soil. The SLLL in Fig. 3b by Leenaars et al.(2018) was created based on AfSoilGrids250 m (Hengl et al., 2015) and different pedo-transfer functions. They used pedo-transfer functions particularly developed for tropical soils that need input data e.g., bulk density, cation exchange capacity and pH besides the other soil properties that we used in this study (i.e., sand, silt, clay and organic carbon content). In general, the SLLLs from AfSIS-GYGA are slightly higher than what we produced. This may be due to the different input soil database at different resolution (AfSoilGrids250 m vs. SoilGrids1km) and different pedo-transfer functions used. However, a permanent wilting point of 0.44 cm^3^ cm^−3^ in Ethiopia (Fig. 3b) seems to be too high in reality from AfSYS-GYGA. Overall, the spatial distributions of SLLLs are comparable (Fig. 3).

**Figure A.3:**
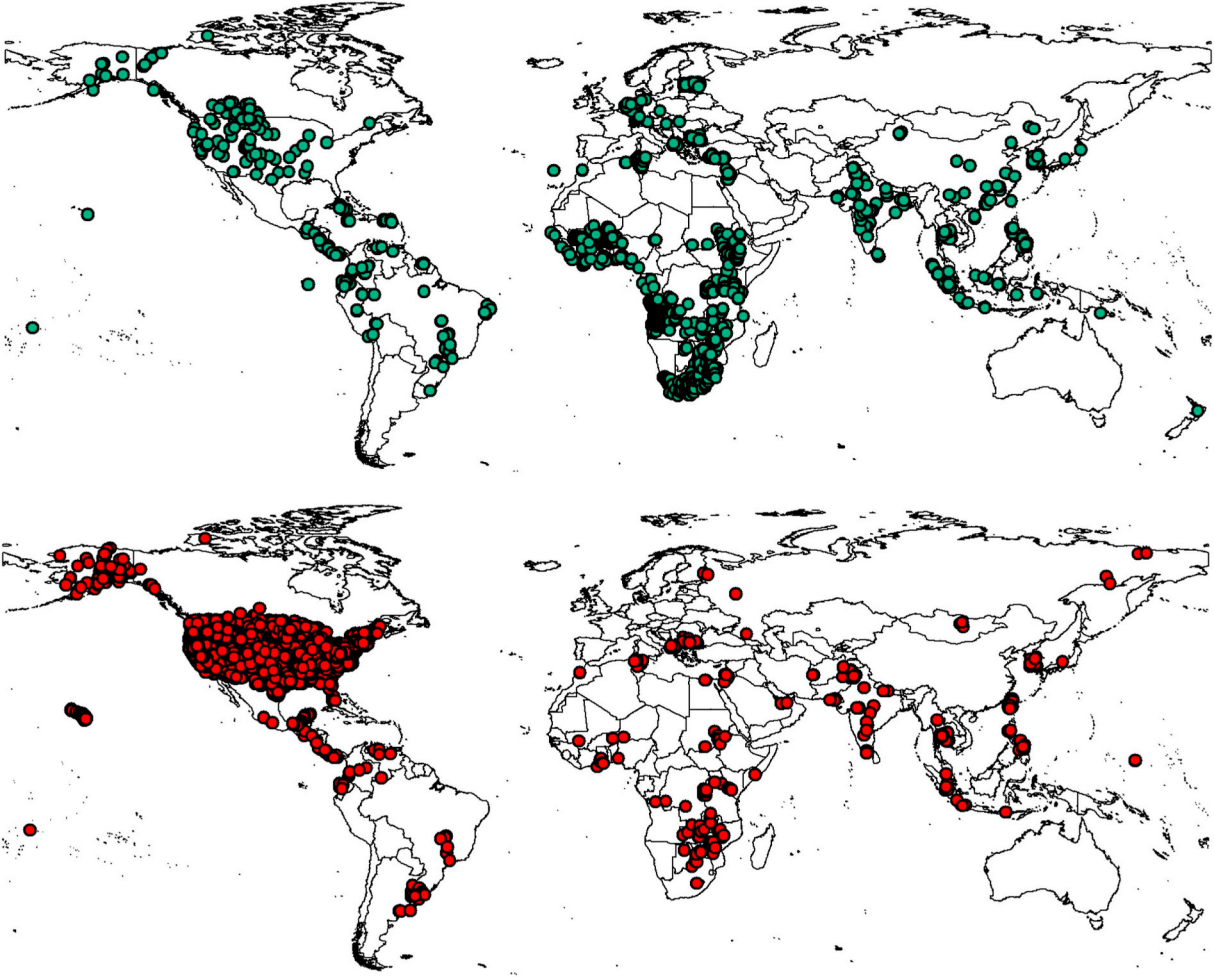
Locations of WoSIS soil profiles which have soil water content at wilting point (top) and at field capacity (bottom) in soil depth up to 15 cm (from the “July 2016” WoSIS data downloaded in August 2018)

The soil water contents at wilting point (SLLL) and field capacity (SDUL) of the new soil profile dataset (this study) were compared with ISRIC WoSIS database; ISRIC WoSIS contains standardized soil profile data collected by many international soil data providers. For a fair comparison, we filtered out data from ISRIC WoSIS to remove soil properties of depths deeper than 15 cm. The locations of WoSIS SLLL and SDUL soil profiles can be found in Fig. A3 (Appendix). A total of 8643 SDUL values were compared, but 96% of them were from the continental U.S. In the case of SLLL, however, data from the U.S. only takes 6.2% of total 1662 SLLL values. In the comparison, SDUL data greater than 0.95 cm^3^cm^−3^ were excluded from WoSIS.

**Fig. 4:**
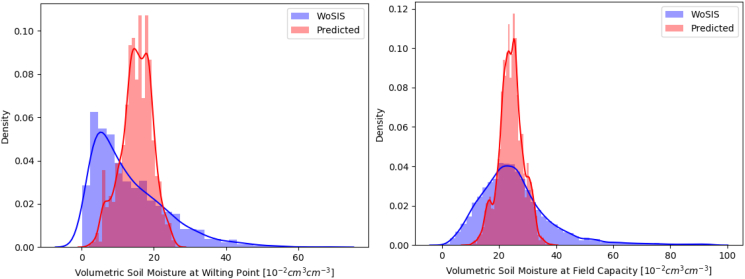
Kernel density plots of soil water content at wilting point (left) and at field capacity (right). Blue and red colors represent WoSIS data and soil properties derived in this study, respectively. (For interpretation of the references to color in this figure legend, the reader is referred to the Web version of this article.)

Note that there are scale discrepancies between the WoSIS data and our derived SLLL and SDUL (see Section 3.2). Instead of calculating error statistics, we compared the kernel density plots of the two soil parameters (SLLL and SDUL) from WoSIS and the parameters we produced from this study (Fig. 4). The SLLL values from WoSIS are more positively skewed and have a lesser mean compared with the SLLLs we derived. The distributions of both SLLL and SDUL produced in this study are narrower than the ones from WoSIS. This could be due to the smoothing effect when values are aggregated at 10-km scale. The pedo-transfer functions applied in this study may have also partially contributed to the narrower distribution. However, considering that crop simulation models will be highly likely applied to areas suitable for agriculture, the SDUL or SLLL values in extreme ranges (too low or high, such as SDUL greater than 0.6 cm^3^cm^−3^) are not appropriate to be used for evaluation. In general, the SLLL and SDUL values we derived from this study are within reasonable ranges for agricultural soils.

#### 4.1.2 Available water content

**Fig. 5:**
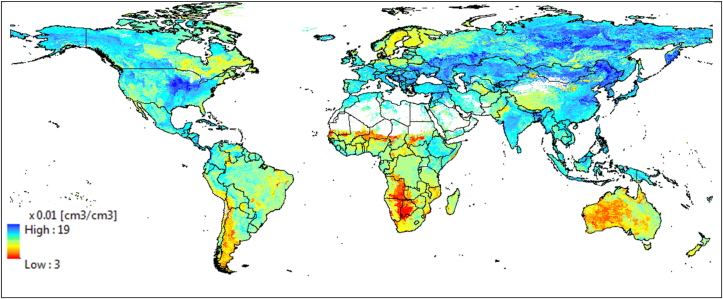
Derived AWC for the first soil layer (0–5 cm).

Fig. 5 shows the available water content (AWC) of the first soil layer (0–5 cm) at 10-km resolution. As expected, derived AWC is very low in areas where the world's major deserts are located (e.g., Sahara, Kalahari and Namib in Africa, Pantagonia in South America, Western Australia and Northern India). Derived AWC with a maximum value of 0.19 cm^3^ cm^−3^ seems to be low compared with laboratory measurements, which could be due to the smoothing effect of spatial aggregation. AWC of the 2nd soil layer (5–15 cm) showed a similar spatial distribution as in Fig. 5 (not shown).

**Fig. 6:**
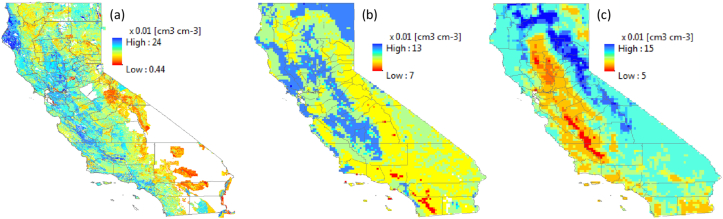
AWC maps of California from SSURGO (a), derived AWC from SoilGrids1km (b), aggregated AWC from SoilGrids250 m (c). Note that we used different color scales intentionally to shows the distributions more clearly. (For interpretation of the references to color in this figure legend, the reader is referred to the Web version of this article.)

Due to the difficulties of collecting global AWC maps, we relied on well-documented soil database, SSURGO of the U.S. for additional evaluation of the derived soil hydraulic properties. The gridded SSURGO (gSSURGO, Soil Survey Staff, 2014) provides a wide range of soil information including available water storage (mm) of top 25 cm of soil depth. An example in California (USA), The AWC values derived from SoilGrids1km (SDUL minus SLLL) at 10-km resolution in this study (Fig. 6b) were compared with AWCs from SSURGO in Fig. 6a and weighted averages of AWCs at an aggregated 10-km resolution from SoilGrids250 m (Hengl et al., 2017) in Fig. 6c. Note that AWCs in Fig. 6b and c represents value at 15 cm soil depth through weighted averages of two standard soil layers (0–5 and 5–10 cm), while the SSURGO-AWC values in Fig. 6a represent 25 cm of soil depth. AWCs from SoilGrid1km have a narrower range of distribution (i.e., maximum value of 13 and minimum value of 7) compared to SSURGO. This narrow range may be attributed to the narrower range of SDUL distribution as shown in Section 4.1.1 in comparison with WoSIS. Other reasons could be the aggregation effect and the PTFs applied. Gijsman et al. (2002) compared 8 different methods to derive soil hydraulic properties and demonstrated that the estimated AWC values vary significantly, “not only between methods and soil types, but just as well within soil types”. Therefore, it is recommended to test sensitivities of the selected PTFs in future studies. Despite of the narrower ranges in the magnitudes of AWCs, the AWCs derived from this study show a very similar distribution with SSURGO i.e., major agricultural areas along the Great Valley have relatively higher AWC values while forest areas along the Sierra Nevada have the lower values. Interestingly, AWC map from SoilGrids250 m shows an opposite distribution, between SSURGO, and this study.

**Fig. 7:**
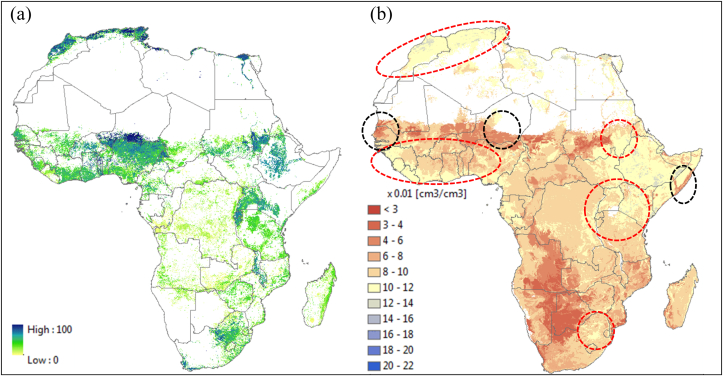
Comparison of derived available water content (AWC) with other dataset: (a) IIASA-IFPRI cropland percentage map, (b) AWC derived from SoilGrids1km at soil depth 5 cm. Red circles indicate that relatively higher AWC values correspond to the areas with higher percentage crop land while black circles shows opposites. (For interpretation of the references to color in this figure legend, the reader is referred to the Web version of this article.)

As an indirect evaluation of the derived AWC from this study, a cropland map was compared with the derived AWC map of Africa. We used the most recently released global cropland percentage map developed by the International Institute for Applied Systems Analysis (IIASA)- International Food Policy Research Institute (IFPRI) (Fritz et al., 2015). Fig. 7 suggests that many cropland areas in Africa are consistent with areas of relatively higher AWC values (Fig. 7b; see red circles). However, there are also some areas that do not match, as indicated in black circles (Fig. 7b).

As shown in Section 4.1.1, we also compared the derived AWC with another derived AWC map from AfSIS-GYGA (Leenaars et al., 2018). The AfSIS-GYGA's AWC map (see Fig. 6b of Leenaars et al., 2018) shows a somewhat different spatial distribution from our results in Fig. 7, specifically for relatively higher AWC regions throughout the savanna zone in Africa and very low AWC in the Gezira, Sudan (not shown here, see Han et al., 2015). Leenaars et al. (2018) attributed the strange patterns in their results to the limited availability of bulk density data.

### 4.2 Case study: ex-ante assessment of maize yield response potential to fertilizer, hybrid seeds, and improved agronomy

**Fig. 8:**
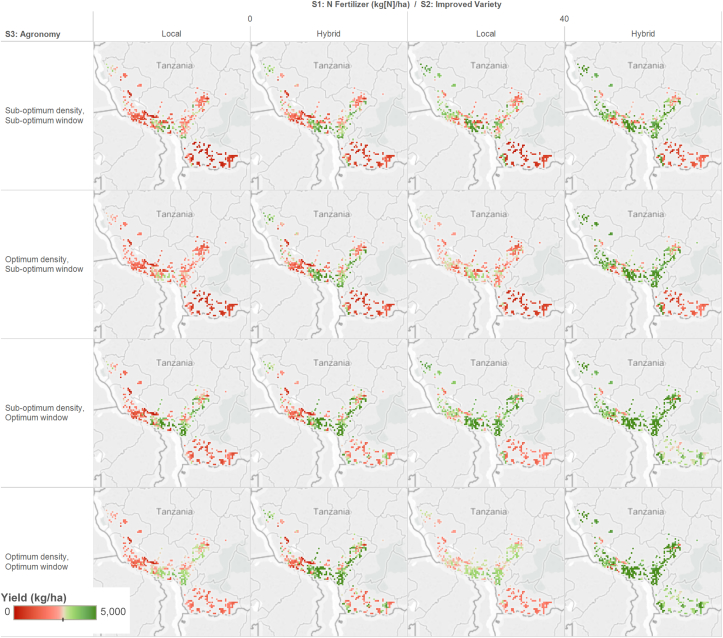
Simulated maize yield responses to three step-wise intensification strategies (fertilizer, variety, and agronomy). Note that yields were capped at 5 t/ha.

This section shows an example of how the new gridded soil profile database can be used for real-world applications. Southern highlands area in Tanzania is often referred to as one of the breadbasket in the region, yet smallholder farmers’ productivity is still very low. Latest national statistics data from FAOSTAT shows the national average yield of maize as 1.3 t ha^−1^ in 2013, with annual growth rate of 2.71%. To assess how much maize productivity increases can be achieved by future investment on intensification options, a grid-based crop-modeling framework was developed using the SoilGrids1km-based soil profiles and used for each 10-km grid cell in the area. From the calibrated baseline yield levels with subsistence farming system, where farmers use traditional variety with no fertilizer application with inadequate agronomic information, three step-wise strategies were simulated: 1) increased nitrogen (N) fertilizer application, 2) improved variety, and 3) optimum planting window. Specifically, the ex-ante study aimed to identify the feasibility of achieving the target yields of 3 t ha^−1^ from the simulated intensification strategies. Simulation results showed, overall, that the target yield level could be readily achieved through the intensifications with agronomic interventions (e.g., optimum planting density and planting window), spatial variability of yield response potentials were demonstrated across the region in Fig. 8.

## 5 Discussion

This study presents a new global gridded soil profile dataset at a 10-km resolution developed for DSSAT. Despite recent advances in digital soil mapping, there is still a gap in utilizing the available soil databases for crop models, and eventually for practical decision-making by policy makers for food security under a changing climate, and environment. The gap is mainly because of the models’ required soil inputs that are not provided by typical soil survey data. Especially for gridded crop model applications, availability of soil input data at the right format and resolution has been one of the big challenges. In this study, we bridge this gap by developing a 10-km global gridded soil profile dataset by translating a high-resolution gridded soil database, SoilGrids1km, into model-ready formats and specificity. This work aligns with Carré et al. (2007) and McBratney et al. (2012) that asserted the concept of “digital soil assessments” beyond digital soil mapping. Linking crop models with soil inputs is a critical step in assessing biomass production (e.g., crop yield) as one of the important soil functions, as well as bringing the value of digital soil maps to benefit society.

The soil profile dataset developed in this study has been used operationally since its first release in 2015. Andreadis et al. (2017) developed the Regional Hydrologic Extremes Assessment System (RHEAS) and coupled it with DSSAT to monitor and predict drought and maize productivity in East Africa. RHEAS used the gridded soil profile data from https://doi.org/10.7910/DVN/1PEEY0 for regional DSSAT modeling. Komarek et al. (2018) applied a gridded DSSAT using the soil profiles of Han et al. (2015) to simulate and analyze economic effects of different seed cultivars and fertilizer intensification in Tanzania. The developed soil profile dataset as it is now has a great potential to be used for developing agriculture-related decision support tools or any analysis framework at regional or global scale for researchers or policy makers. The methods described in this study and Han et al. (2015) can be used as precedence to developing other gridded soil datasets for other crop models like Agricultural Production Systems sIMulator (APSIM) or Environmental Policy Integrated Climate (EPIC) model.

In this study, we applied the pedo-transfer function of Saxton and Rawls (2006) to derive the required soil hydraulic properties for DSSAT. However, there are other pedo-transfer functions that can be used for this purpose. Likewise, there are several ways to estimate soil properties that are not available from SoilGrids1km to complete the model-friendly soil profile dataset, although here we used the generic soil profiles in HC27. These are potential sources of uncertainties in the dataset. Further work is needed to quantify uncertainties and sensitivities of the applied methods to the final product.

Uncertainty quantification has received a great attention particularly by the GlobalSoilMap community (Heuvelink, 2014) because of its importance to the end-users as well as to the map makers. Uncertainties of the derived soil profile dataset can come from different sources. Moreover, additional uncertainties can come also from subsequent processes when applying pedo-transfer functions, and when aggregating the data from 1-km to 10-km resolution. Han et al. (2015) earlier quantified uncertainties of derived soil hydraulic properties based on the assumptions of independence from each pixel or normality of soil properties following Heuvelink (1999) and Hengl et al. (2014). Assumptions like this may not be always valid in realistic. More sophisticated approach for quantifying uncertainty can be employed e.g., statistical approaches to quantify probability distributions of all possible sources of uncertainties (Heuvelink, 2014). This statistical approach however can be computationally expensive and requires sufficiently large sample size to characterize uncertainties, thus warrants as a separate study.

The product of this study has a final resolution of 10-km derived from a 1-km resolution dataset. The question of how to aggregate data from 1-km to 10-km arises. This is related to defining the “support” of the soil properties. An approach to finding a representative soil property at a 10-km resolution is to select a dominant soil pixel/profile in the target 10-km grid as Wu et al. (2010). However, selecting a dominant soil type is not always the best approach. Heuvelink (2007) and Lagacherie et al. (2000) argued that selecting a representative profile of a dominant mapping unit can lead to neglect a substantial part of the mapping unit. In addition, soil covariates used for interpolating point measurements are not always from a dominant soil profile. Hengl et al. (2017) applied a digital elevation model and a wide range of remote sensing data (e.g., MODIS-Enhanced Vegetation Index) as soil covariates to creating soil database at 250-m resolution. These gridded soil covariates are representative values, and not necessarily from a dominant soil type of the pixel.

In this study, we employed an arithmetic averaging of soil properties to aggregate 1-km resolution data to a 10-km resolution. The reasoning behind using the simple average is that, we assumed that our target pixel is a homogeneous unit, having a hypothetical representative soil property, which may be neither a simple average of a few ground-measured values, nor values from one dominant soil profile when we apply DSSAT to a grid of a 10-km resolution. This is similar to the approach of Mohanty and Zhu (2007). Regarding the issue of defining a representative value for a grid cell, Shangguan et al. (2014) addressed the problem of finding “one to one” relation between a grid cell and soil mapping units (in reality there are “one to n” relations between a soil mapping unit or soil map polygon or a grid). They tested three methods: area-weighted, dominant soil type and dominant binned soil attribute method, but offered results of the areas-weighted method (which is similar to our simple averaging of 1-km pixel values). Simple averaging approach has been used commonly in remote sensing community but not in soil science community. Further investigation is needed to better understand the effects of different aggregation schemes.

## 6 Conclusions

This study developed a set of DSSAT compatible global soil profiles at 10-km grid based from a recently released soil database, SoilGrids1km. Based on the soil properties from SoilGrids1km, soil hydraulic properties were derived using pedo-transfer functions. Other required variables were derived from HarvestChoice's HC27 dataset. The final product is provided for each country in a *.SOL file, which is a standard format of the DSSAT soil input.

Since it is not possible to have a reference data close to “true” values representing a 10-km resolution, our evaluation of the output soil dataset had to rely on more indirect methods, qualitative approach rather than quantitative. Visual inspections were conducted in comparison with i) other maps (i.e., IIASA-IFPRI cropland map and AfSIS-GYGA's wilting point and available water content maps) and ii) SSURGO soil database for California in the U.S. In general, distributions of the soil properties (areas with relatively higher or lower values) matched well with the data in comparison. When the output was compared with WoSIS soil database, which contains global harmonized soil profiles, smoothing effect due to the aggregation was observed. As an application, the soil profile datasets developed in this study was tested in an ex-ante modeling study to assess the potential of agricultural investments in Tanzania's southern highlands area.

Uncertainties incurred from the input soil database and during the data processing should be quantified to better assess the quality of the final product. Future work will be done on quantifying the impacts of selected pedo-transfer functions, type of input soil database (SoilGrids250 m rather than SoilGrids1km), and sensitivities of the estimated soil parameters on the crop yield predictions.

The soil profile dataset developed in this study will contribute to the advances of gridded crop modeling applications at regional or global scale. The approach used in this study can be easily applied for other soil databases e.g., SoilGrids250 m, for gridded application of crop models at higher resolution.

## Data availability

Name of data set: Global High-Resolution Soil Profile Database for Crop Modeling Applications.

Developers: International Research Institute for Climate and Society, Columbia University, NY, 10964, USA; Michigan State University, East Lansing, MI, USA; HarvestChoice, International Food Policy Research Institute (IFPRI), Washington D.C., USA.

Contacts: Eunjin Han, International Research Institute for Climate and Society, Columbia University, NY, 10964, USA. email: eunjin@iri.columbia.edu Amor VM Ines, Michigan State University, MI, USA. email: inesamor@msu.edu.

Jawoo Koo, International Food Policy Research Institute (IFPRI), Washington D.C., USA. email: j.koo@cgiar.org.

Year first available: 2015.

Availability: The soil dataset product is available at https://doi.org/10.7910/DVN/1PEEY0 under a Creative Commons CC-BY-NC (Attribution, Non-commercial) 4.0 license via Dataverse.

Fortran codes for deriving soil parameters are also freely available via GitHub (https://github.com/Agro-Climate/Global-gridded-soil-data-for-DSSAT-at-10km).

## Figures and Tables

**Figure A.2:**
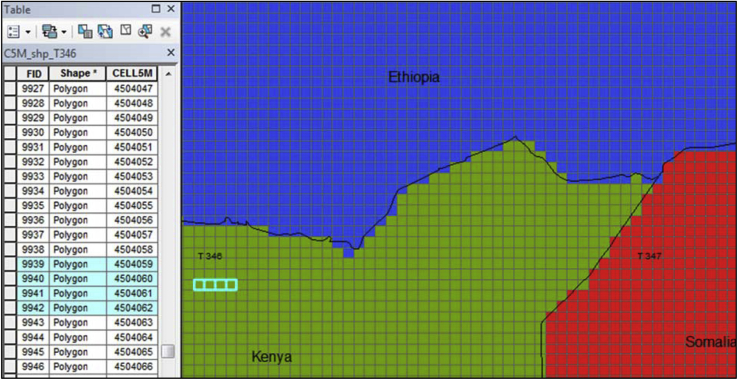
HarvestChoice's standard grid-cell ID (CELL5M) and corresponding country masks over Kenya, Ethiopia and Somalia.

**Table A.1:** Short names for soil texture classifications

Soil texture classification	Short name
Silt loam	SiltLoam
Sand	SAND
silty clay loam	SiltClayL
Loam	Loam
clay loam	ClayLoam
sandy loam	SandyLoam
silty clay	SiltyClay
sandy clay loam	SandClayL
loamy sand	LoamySand
Clay	CLAY
Silt	SILT
sandy clay	SandyClay

Except for four soil hydraulic properties (SLLL, SDUL, SSAT, SSKS) derived from PTFs, other unknown soil properties are extracted from HC27, as described in Table 2. Based on the given classified soil texture, root depth and fertility (organic carbon), one soil profile out of 27 profiles was selected. The only exception is for SLMH (Master horizon) as it was pre-determined for each standardized soil layers so that it can be similar with HC27. For the first two layers, ‘A’ is assigned and ‘AB’, ‘BA’, ‘B’,’BC’ are assigned to the rest 3rd to 6th layers, respectively.

## APPENDIX

### A.1. Data Processing for Output Format

DSSAT-compatible soil profile data (*.SOL) were saved separately for each country. The file names were derived from two-letter standard country code (ISO_A2) of ISO (International Organization for Standardization). For example, KE.SOL file contains all available soil profiles at 10-km resolution in Kenya. Two different country codes, ISO_A2 and ISO_A3 (three-letter code) were also written in each profile as shown in the line 1 (KEN) and 3 (KE) in Fig. A1. Complete list of ISO country code and corresponding country boundary map was obtained from Natural Earth (http://www.naturalearthdata.com/).

Soil profile ID (e.g., KE04430591 in Fig. A1) is determined from HarvestChoice's standard grid-cell ID at 10-km spatial resolution, CELL5M (HarvestChoice and Minnesota, 2018). Fig. A2 shows a sample of CELL5M grid-cell IDs across Kenya as an example. Naming soil profile ID based on CELL5M IDs is intended to streamline grid-based crop modeling operation across large areas in a batch within the HarvestChoice's modeling framework.

Creating country mask at 10-km resolution is not easy due to mismatches between curved country boundaries or coastal lines and 10 km grids as shown in Fig. A2. We used IFPRI's concordance look-up table to assign a country ID for each 10-km grid (HarvestChoice and Minnesota, 2018).

Soil texture classification in the first line of *.SOL (e.g., SandClayL in Fig. A1) is determined based on the proportion of sand and clay in a given grid using USDA-SCS soil triangle (Gerakis and Baer, 1999). Due to limitation in space, the soil texture classification was truncated to shorter names when needed (see Table A1).
